# The effect of probiotic supplementation on non-alcoholic fatty liver disease (NAFLD) fibrosis score in patients attending a tertiary hospital clinic in Cairo, Egypt

**DOI:** 10.1186/s12876-024-03424-3

**Published:** 2024-10-08

**Authors:** Alaa Ahmed Abd El Hamid, Azza Emam Mohamed, Manal sabry Mohamed, Ghada Essam El-Din Amin, Hagar Ahmed Ahmed Elessawy, Mohamed Farouk Allam

**Affiliations:** 1 Department of Family Medicine, Faculty of Medicine, Ain Shams University, Cairo, Egypt; 2 Gastroenterology and Hepatology, Internal Medicine Department, Ain Shams University, Cairo, Egypt; 3 Department of Community, Environmental and Occupational Medicine, Faculty of Medicine, Ain Shams University, Cairo, Egypt

**Keywords:** Non-alcoholic fatty liver disease, Non-alcoholic steatohepatitis, Probiotics, NAFLD fibrosis score

## Abstract

**Background:**

Non-alcoholic fatty liver disease (NAFLD) is characterized by hepatic fat accumulation (> 5% of liver tissue) in the absence of alcohol abuse or other chronic liver diseases. NAFLD can progress to non-alcoholic steatohepatitis (NASH), fibrosis, cirrhosis, and hepatocellular carcinoma (HCC). This study aimed to assess the efficacy of probiotic (lactobacillus) supplementation on NAFLD fibrosis score.

**Methodology:**

A double-arm randomized controlled trial was conducted in the family medicine clinic of a tertiary hospital, enrolling patients with sonographic evidence of NAFLD. Fifty patients were divided into two groups: the Probiotic group received lifestyle modification instructions along with daily probiotic supplementation for twelve weeks, with regular monthly follow-up visits. The Standard Treatment group received low-fat diet and lifestyle modification instructions only.

**Results:**

The mean age of participants was 46.10 years (SD 10.11), with 70% females and 30% males. The study found a statistically significant difference in liver enzymes (ALT and AST) and BMI in the probiotic group before and after intervention. However, there was no significant difference in NAFLD fibrosis score between the two groups.

**Conclusion:**

Short-term probiotic treatment resulted in improvements in ALT, AST, and BMI in the probiotic group, but did not significantly affect NAFLD fibrosis score. Further research with larger sample sizes and longer follow-up periods is warranted.

**Trial registration:**

The clinical trial was registered at Protocol Registration and Results System with number NCT06074094 (12/09/2021).

## Background

Non-alcoholic fatty liver disease (NAFLD) is characterized by the accumulation of fat in more than 5% of liver tissue, occurring in the absence of alcohol abuse or other chronic liver diseases. NAFLD can lead to liver inflammation and may progress to non-alcoholic steatohepatitis (NASH), fibrosis, cirrhosis, and hepatocellular carcinoma (HCC) [1].

The prevalence of NAFLD varies between 19% and 33% across different populations, influenced by factors such as ethnicity, region of origin, obesity, and the presence of other conditions like diabetes mellitus or hypertension [2].

The prevalence of NAFLD among Egyptian young adults was estimated to be 31.6% [3], closely matching the 31.8% prevalence rate found in a meta-analysis of several epidemiological studies among the general Middle Eastern populations [4].

NAFLD imposes a significant economic burden and negatively affects health-related quality of life [5]. Longitudinal studies have shown that NAFLD increases all-cause mortality, cardiovascular mortality, and liver-related mortality [6]. It is projected to become the most common reason for liver transplantation globally by 2030 [7]. Patients with NAFLD experience higher overall mortality, with most deaths resulting from cardiovascular events [8].

Many individuals possess risk factors associated with NAFLD, making it crucial to identify those at high risk to prevent disease progression [9]. Major risk factors for increased NAFLD prevalence include being male, older age, specific ethnicities, hypertension, hypercholesterolemia, diabetes mellitus, lower socioeconomic status, lower educational attainment, and reduced physical activity [10, 11]. Type 2 diabetes mellitus (T2DM) is particularly significant, as it more than doubles the risk of developing severe liver disease [9].

The combination of intestinal dysbiosis and gut-derived microbial products may significantly contribute to the development of chronic inflammatory conditions such as NAFLD and NASH [12].

Currently, no drug is registered for the treatment of NASH. Although lifestyle management is commonly recommended, it can be challenging to sustain [13].

The primary challenge in clinical practice is identifying patients with advanced liver fibrosis or cirrhosis, who are at the highest risk for complications [14]. Liver biopsy, despite its diagnostic accuracy, is impractical and unsuitable due to the large number of high-risk patients and its inherent limitations, particularly in primary care settings [15].

The NAFLD Fibrosis Score (NFS) is a validated, noninvasive tool used to identify patients whose NAFLD has progressed to liver fibrosis [16]. For patients with a high NFS, further testing may be warranted, including Enhanced Liver Fibrosis (ELF) score, elastography, or liver biopsy [17].

Patients diagnosed with NAFLD should undergo an initial assessment of liver fibrosis using NAFLD scores. Primary care clinics can effectively manage follow-up for patients whose NFS is below − 1.455 [18].

The current standard of care for treating NAFLD emphasizes lifestyle interventions, particularly through diet and exercise [19]. European guidelines recommend the Mediterranean dietary pattern specifically for NAFLD treatment [20]. Statins are also recommended to lower cardiovascular risks and are generally safe for most NAFLD patients [21]. Additionally, insulin-sensitizing agents like metformin or thiazolidinediones are used to reduce insulin resistance [20]. These strategies not only target NAFLD management but also address associated cardiovascular risks, given their shared metabolic implications.

Vitamin E is recognized for its ability to reduce oxidative damage to liver cells [22]. Sodium-glucose co-transporter inhibitors (SGLT2i) may alleviate steatosis through several mechanisms, including moderate weight loss, improved glycemic control, reversal of hepatic insulin resistance, and enhanced glucose sensitivity of pancreatic beta-cells [23]. These treatments offer promising avenues for addressing NAFLD by targeting specific pathways involved in liver health and metabolic regulation.

Microbial therapies encompass treatments such as prebiotics and probiotics, which aim to manipulate the intestinal *microbiota*. Prebiotics are non-digestible food ingredients that foster the growth of beneficial microorganisms in the intestine [24]. Meanwhile, probiotics work by modulating the gut *microbiota*, thereby reducing pathogenic bacteria, enhancing gut barrier integrity, and mitigating liver inflammation [25]. These therapies represent innovative approaches to managing conditions like NAFLD by targeting the gut-liver axis and influencing overall gut health.

The objective of the present study was to evaluate the effectiveness of probiotics supplementation (lactobacillus) on NAFLD fibrosis score.

## Methodology

### Study design and setting

The study was a double armed randomized controlled clinical trial conducted in family medicine and internal Medicine clinic at tertiary hospital in Cairo, Egypt.

### Inclusion criteria

age 18–60 years, with body mass index equal or more than 25 kg/m2 and sonographic findings of NAFLD.

### Exclusion criteria

any evidence of other chronic liver diseases including viral hepatitis (B and C), auto immune hepatitis, history of alcohol drinking…etc., history of DM, history of statin therapy for cardiovascular disease (CVD), history of recent operation, history of gastroenteritis or diarrhea in the last 2 week were excluded.

### Sampling and study participants

Participants were randomly selected from Family Medicine and internal Medicine Outpatient clinics at Ain Shams University Hospitals in Cairo, Egypt. A sample size of 25 participants in each group of probiotic and standard treatment group was estimated.

### Sample size estimation

The sample size was determined based on a recent study by Famouri et al. (2017) conducted at the Pediatric Clinics of Isfahan University of Medical Sciences, Iran. The calculations were made with a 95% confidence level and 80% power. The study anticipated that 53% of patients treated with probiotics would have a normal liver confirmed by ultrasound, compared to 16.5% of those treated with a placebo. The initial sample size per group was calculated to be 21. Accounting for a 15% expected loss to follow-up (approximately 3 patients per group), the final sample size was adjusted to 24, rounded up to 25 per group. The sample size was calculated using EPIDAT software version 4.1.

### Grouping

**Probiotic group** received lifestyle modification instructions in addition to probiotic supplementation (^®^Lactèol Fort 10 billion) once daily for twelve weeks and regular follow-up visit every month. Lactèol Fort Sachet Powder contains Culture Medium and Lactobacillus Lb as its active ingredients. We chose “Lacteol Fort” because it is reasonably priced and readily available in Egypt.

**The standard treatment group** followed non-pharmacological measures including dietary control with low-fat intake and mild to moderate regular physical exercise twice weekly.

Non-pharmacological measures were recommended to both groups.

We applied a standard recommended diet with macronutrient distribution ranges of 45–65% of daily calories from carbohydrates, 20–35% from fats, and 10–35% from protein.

#### Randomization

The researcher used sealed envelopes containing code for intervention or control.

All participants were referred for performing liver ultrasonography. After including all eligible subjects to the study, the subjects were randomly assigned to one of the two groups receiving probiotic or standard treatment group.

For subjects in the probiotic group, one daily probiotic sachet which contains culture medium and lactobacillus LS as active ingredients was administered for 12 weeks with regular follow-up visit every month.

Healthy lifestyle habits were recommended for participants of both groups; they were encouraged to increase their daily activity, as well as to improve their dietary habits by increasing the intake of fruits and vegetables, and by decreasing the consumption of fast foods, high-fat meals, processed food, and sweet drinks.

At the beginning and that end of the trial, anthropometric examination and laboratory measurements were performed for all participants. Weight and height of participants were measured, BMI was calculated as weight (kilograms) divided by height in square meter (meter square). Waist circumference (WC) was measured by a non-elastic tape at a point midway between the lower border of the rib cage and the iliac crest at the end of normal expiration.

Venous blood sample was taken and Serum alanine aminotransferase (ALT), aspartate aminotransferase (AST), complete blood count (CBC) and Albumin were measured before the intervention and after twelve weeks of intervention, NAFLD fibrosis score was calculated before and after the intervention.

### Ethical considerations

The study protocol was reviewed by the administrative and ethical committee board approvals at Ain Shams University Hospital (IRB number MD 88\2020). The clinical trial was registered at Protocol Registration and Results System with number NCT06074094 (05/10/2023). An informed Consent was obtained from all participants before starting interviews. This study was executed according to the code of ethics of the World Medical Association (Declaration of Helsinki) for studies on humans.

### Statistical analysis

The collected data were introduced and statistically analyzed by utilizing the Statistical Package for Social Sciences (SPSS) version 26 for windows. Qualitative data were defined as numbers and percentages. Chi-Square test, Fisher’s exact test and McNemar test were used for comparison between categorical variables as appropriate. Quantitative data were tested for normality by Kolmogorov-Smirnov test. Normal distribution of variables was described as means and standard deviation (SD) or median and ranges, and Student’s t test, and Mann-Whitney U test, Paired t-test and Wilcoxon signed-rank test were used for comparison between groups. P value ≤ 0.05 was statistically significant.

## Results

Initially, 50 patients with NAFLD were enrolled in the study and allocated into 2 groups probiotic (*n* = 25) and standard treatment group (*n* = 25). Baseline characteristics of patients in the 2 study groups are presented in Table 1. The groups were matched regarding age, sex, weight, BMI, and waist circumference.

The mean age of the whole studied sample was 46.48 and the number distribution of gender among all subjects was 15 (30%) male and 35 (70%) female. There was no statistically significant difference between the standard treatment group and the probiotic- treated group regarding smoking, weight, and BMI.

**Table 1:** Baseline demographic and anthropometric characteristics in patients with NAFLD

Variable		Probiotic group (*N* = 25)	Standard treatment group (*N* = 25)	*P* value
Age	Mean (SD)	45.72 (8.9)	46.48 (11.60)	0.793
Gender	Male	7 (28%)	8 (32%)	0.758
	Female	18 (72%)	17 (68%)	
Weight (kg)	Mean (SD)	89.84 (15.64)	87.84 (13.33)	0.629
BMI (kg\m²)	Mean (SD)	33.88 (7.43)	31.21 (3.32)	0.108
WC	Mean (SD)	109.56(10.21)	104.40 (9.24)	0.067

BMI: body mass index; WC: waist circumference

As shown in Table 2 the median ALT, AST, Albumin, and platelet were not statistically different between probiotic treated group and standard treatment before and after the intervention There was a significant decrease in ALT and AST in the probiotic-treated group (P values 0.014 and 0.004, respectively) and a smaller decrease in the standard treatment group in AST only (P value 0.025).

There was no significant difference between the two groups regarding NAFLD fibrosis score before and after intervention.

**Table 2:** Median metabolic parameters at baseline and three months later in studied groups

Variable		probiotic group (*N* = 25)		Test Value	P-value	Standard treatment group (*N* = 25)		Test value	P-value
		Pre-intervention	Post-intervention			Pre- intervention	Post- intervention		
**ALT**	Median (IQR)	23 (18–33)	21 (13–26)	-2.464≠	0.014	20 (16–26)	20 (16–24)	-1.509≠	0.131
	Range	8–73	8–73			10–38	8–35		
**AST**	Median (IQR)	22 (18.3–35)	18 (14–21)	-2.895≠	**0.004**	21 (19–25)	21 (18–25)	-2.249≠	**0.025**
	Range	12–80	11–59			14–48	12–40		
**albumin**	Mean (SD)	4.30 (0.29)	4.22 (0.35)	1.060	0.300•	4.36 (0.36)	4.29 (0.22)	1.534•	0.138
	Range	3.9–5	3.5–4.9			3.45–4.9	3.9–4.7		
**Platelet**	Mean (SD)	304.76 (52.07)	306.12 (60.93)	-0.122	0.904•	286.88 (57.79)	283.36 (58.39)	0.510•	0.615
	Range	180–389	181–429			161–410	170–390		
**NAFLD** **fibrosis score**	Median (IQR)	-3.01 (-3.46 – -1.79)	-2.71 (-3.65 – -1.61)	-0.578≠	0.563	-2.43 (-3.43 – -2.08)	-2.27 (-3.39 – -1.6)	-1.117≠	0.264
	Range	-4.13–0	-4.42–0.22			-4.88 – -0.51	-5.09 – -0.52		

•: Paired t-test; ≠: Wilcoxon Ranks test.

Table 3 Shows that there was no statistically significant difference between two groups regarding fibrosis before and after intervention. However, from the baseline to the end of the study, the probiotic- treated group, there was a decrease in fibrosis from intermediate score to absence of significant fibrosis.

**Table 3:** Fibrosis at baseline and three months after the intervention

		Test of significance		Test of significance		
Variable		Probiotic group (*N* = 25)	Standard treatment group (*N* = 25)			
				Test value	*P*-value	Sig.
Fibrosis pre	Absence of significant fibrosis (F0-F2)	19 (76%)	22 (88%)	Fisher’s Exact test	0.463	NS
	Indeterminate score	6 (24%)	3 (12%)			
Fibrosis post	Absence of significant fibrosis (F0-F2)	20 (80%)	20 (80%)	X^2^ = 0.00	1.00	NS
	Indeterminate score	5 (20%)	5 (20%)			
	McNemar test (p-Value)	1.0 (NS)	0.5 (NS)			

## Discussion

Probiotics are considered a promising treatment option for NAFLD since they are inexpensive, readily available, and do not cause significant adverse effects. However, there is still a debate on their use. Probiotic use has really been regarded as a viable and promising method of controlling the gut flora [26, 27]. In pathological processes involving NAFLD and its variants, probiotics can be used as an adjuvant therapy due to its role in enhancing the intestinal barrier, inhibiting the production of metabolites harmful to the liver in addition to influencing the immune system [27].

Most of the study participant were middle aged with mean age 46.10 (SD 10.11), and the mean age of the two studied groups was nearly the same in the probiotic and standard treatment group 45.72 (SD 8.59) and 46.48 (SD 11.60) respectively this agrees with previous studies which reported that NAFLD is more prevalent in middle and elderly population as risk factors for developing NAFLD tend to become more prevalent as age increases [34].

In the probiotic group after twelve weeks of intervention the weight significantly decreased from 89.84 (SD 15.64) to 87.60 (SD 15.26) (*P* = 0.004), BMI from 33.88 (SD 7.43) to 33.04 (SD 7.35) [*P* = 0.002], and WC decreased from 109.56 (SD 10.21) to 108.28 (SD 9.98) [*P* < 0.001], so in this study probiotics lower body weight in agreement with a study conducted in 2014 by ***Nabavi et al.*** [28] that demonstrated that probiotic administration lower body weight.

The probiotic treated group mean levels of liver enzymes (ALT and AST) significantly decreased; this finding was consistent with ***Monem*** [29] who conducted a study at Zagazig University Hospitals in Egypt to assess the impact of probiotics on the outcome of NASH. they attributed the decrease in liver enzymes to the improvement occurred in the inflammatory status of patients after using probiotics. the metabolic products generated by the restructured microbiota or the absorption of distinct bacterial fragments, which have a positive effect on the patients’ metabolism. Meanwhile, ALT did not significantly change in the standard treatment group, and AST only decreased; however, its level is not specific to liver injury and is affected by many other factors. In addition, two double-blind and placebo-controlled studies, shown an important decrease in liver enzymes (ALT, AST), triglycerides, LDL-C, with supplementation of probiotics in obese children [30]. These positive outcomes clearly show the value of probiotics in NAFLD treatment. But further research is still needed to determine whether probiotics may be used to treat nonalcoholic fatty liver disease.

To the best of our knowledge, the present study is the first trial to investigate the probiotics effect on NAFLD fibrosis score. Only metabolic indicators were assessed; the NAFLD fibrosis score was not evaluated in any other way.

There are previous random control trials using different strains and doses of probiotics that demonstrated some interesting positive effects on ALT, AST, blood glucose, insulin resistance and lipid profile. However, even meta-analysis didn’t prove evidence on the usefulness of probiotics in patients with NAFLD [31–33].

In this randomized controlled clinical trial, there was no benefit of the use of probiotics on the NAFLD fibrosis score. The non-significant result of the short-term probiotic therapy on improving the NAFLD fibrosis score does not necessarily mean the lack of its possible benefit effect on liver inflammation and, subsequently, its progression to NASH.

It is worth mentioning that some score elements, like age, cannot be changed by the intervention; other elements, like albumin, take time for laboratory investigations to detect a change; additionally, diabetes was one of the exclusion criteria because, as the previous guidelines mentioned, some anti-diabetic medications can be used as a treatment option for NAFLD [18]. It was also noted that the baseline values of the liver enzymes (ALT and AST) in the two groups (the probiotic group and the standard treatment group) were within the normal ranges of 8–73 and 12–80, respectively. This raised the question of whether the change might be greater if probiotic therapy was applied to patients who had more severe inflammation and elevated enzyme levels.

However, in 2023 a study was carried out by ***Escouto et al.*** [34] who aimed to evaluate the probiotic supplementation on hepatic fibrosis, inflammatory and metabolic markers, and gut microbiota in NASH patients which found that AST to Platelet Ratio Index (APRI) score decreased over time in the probiotic group. This difference may have been caused by the probiotic group’s longer follow-up period (6 months) and the use of different strains of probiotics (Lactobacillus acidophilus and Bifidobacterium lactis).

Before drawing conclusions from the current study, it is important to consider the main limitations: a relatively small sample size and the fact that it was conducted in a single center.

More studies with larger sample sizes, longer duration of treatment, different strains of probiotics, and wide variability of patients are needed to assess the real benefit of probiotics on NAFLD, as their therapeutic use is not supported by high-quality clinical studies to date.

## Conclusion

Probiotic treatment for 12-week duration lowers BMI and liver enzymes (ALT and AST) levels, but it does not outperform the standard treatment in terms of NAFLD fibrosis score.

## Data Availability

The datasets generated and /or analyzed during current study available from the corresponding author on reasonable request.

## Declarations

### Registry of clinical trials

https://clinicaltrials.gov/study/NCT06074094?cond=Probiotics&locStr=Egypt&country=Egypt&rank=9#study-overview.
